# Using Stereolithographic Printing to Manufacture Monolithic Microfluidic Devices with an Extremely High Aspect Ratio

**DOI:** 10.3390/polym13213750

**Published:** 2021-10-29

**Authors:** Pin-Chuan Chen, Po-Tsang Chen, Tuan Ngoc Anh Vo

**Affiliations:** 1 Department of Mechanical Engineering, National Taiwan University of Science and Technology, Taipei City 106335, Taiwan; james60919@gmail.com (P.-T.C.); vnatuan@hcmut.edu.vn (T.N.A.V.); 2 High Speed 3D Printing Research Center, National Taiwan University of Science and Technology, Taipei City 106335, Taiwan

**Keywords:** stereolithography additive manufacturing, monolithic polymeric microfluidics, high aspect ratio microchannels

## Abstract

Stereolithographic printing (SL) is widely used to create mini/microfluidic devices; however, the formation of microchannels smaller than 500 μm with good inner surface quality is still challenging due to the printing resolution of current commercial printers and the z-overcure error and scalloping phenomena. In the current study, we used SL printing to create microchannels with the aim of achieving a high degree of dimensional precision and a high-quality microchannel inner surface. Extensive experiments were performed and our results revealed the following: (1) the SL printing of microchannels can be implemented in three steps including channel layer printing, an oxygen inhibition process, and roof layer printing; (2) printing thickness should be reduced to minimize the scalloping phenomenon, which significantly improves dimensional accuracy and the quality of inner microchannel surfaces; (3) the inclusion of an oxygen inhibition step is a critical and efficient approach to suppressing the z-overcure error in order to eliminate the formation of in-channel obstructions; (4) microchannels with an extremely high aspect ratio of 40:1 (4000 μm in height and 100 μm in width) can be successfully manufactured within one hour by following the three-step printing process.

## 1. Introduction

Microfluidic devices were first developed for gas chromatography in 1979 [1]. Since then, these technologies have been applied in many fields such as chemical analysis and biology study. The substrate materials used for prototyping microfluidic devices include thermoplastics [2,3,4], polydimethylsiloxane (PDMS) [5,6], glass [7], and paper [8,9]. Additive manufacturing has attracted particular interest for manufacturing microfluidic devices due to the speed of moving from design to product, minimal material wastage, customizability, and ease of implementation [10]. Since its initial commercialization in the 1980s, additive manufacturing has been used in aerospace, jewelry creation, and even architecture. The term additive manufacturing refers to the additive manufacture of solid three-dimensional objects layer by layer under precise digital control. This approach to manufacturing has also been applied to the rapid prototyping of polymeric microfluidic devices without the need for conventional bonding processes such as solvent bonding [11] or thermal bonding [12]. Three types of additive manufacturing have been used to create polymeric microfluidic devices including stereolithography (SL), multi-jet modeling (MJM), and fused deposition modeling (FDM). The pros and cons of each method are clearly explained in a review article by Folch [13]. Piironen et al. used a stereolithographic 3D printer (Formlabs, Somerville, MA, USA) to fabricate tapered microchannels (width from 300 to 500 μm) and tilted microchannels (height from 150 to 1050 μm) with the aim of assessing the biocompatibility of 3D printing materials for cell culturing [14]. Showden et al. used an SL 3D printer to manufacture microchannels 3.5 mm in length (3 mm wide and 192 or 250 μm high) aimed at manipulating and assembling cells under hydrodynamic control and subsequently detecting them via electrochemical changes [15]. Romanov et al. used fused deposition modeling (FDM) to create low-cost microfluidic devices (microchannel diameter = 300 μm to 1 mm) for applications of DNA melting and fluorescence imaging analysis [16].

Au et al. reported that stereolithography printing is a possible approach for the creation of microfluidic devices at sub-100 scales [17]. Gong et al. used a proprietary digital light processor stereolithographic (DLP-SLA) 3D printer with specially developed resin to manufacture tiny microchannels [18]. Their analysis of the optical properties of resin and its effect on the minimum size of flow channels permitted the fabrication of microchannels with a cross-section of 60 × 108 μm. Subsequent revisions to the resin formula allowed them to reduce the projected resolution/pixel to just 7.6 μm, which made it possible to fabricate a microchannel with a cross-section of 18 × 20 μm [19]. Their experimental results revealed that the fabrication of microchannels primarily depends on formulating a resin. 

Overall, additive manufacturing provides a simple and efficient approach to creating polymeric microfluidic devices for chemical and bioanalysis. However, creating a microchannel with dimensions smaller than 500 μm is still very challenging and barely reported [20], primarily because of two obstacles. The first obstacle is the resolution of the common additive manufacturing process, which is not sufficient to fabricate fine and microstructures, and the second obstacle is the clogging of the microchannel due to the z-overcure error. From the reviewed articles listed above, the majority of the research on the fabrication of tiny microchannels (<100 μm) has focused on resin formulation, and relatively little research has been dedicated to the influence of the printing process and strategy on the quality of printed microchannels. In the current study, we propose a novel printing strategy by implementing three steps, including channel layer printing, overcoming the z-overcure error by oxygen inhibition, and roof layer printing, to manufacture microchannels (height = 400 μm and width = 100 μm). Multiple experiments were conducted to understand the influence of these printing parameters on the inner surface quality and dimensional accuracy. Finally, we manufactured a microchannel with an extremely high aspect ratio of 40:1 (height = 4000 μm and width = 100 μm), thereby demonstrating that our three-step printing strategy with stereolithographic additive manufacturing can be used for the rapid and bonding-free manufacturing of monolithic polymeric microfluidic devices.

## 2. Materials and Methods

### 2.1. Materials and Apparatus

**Figure 1:**
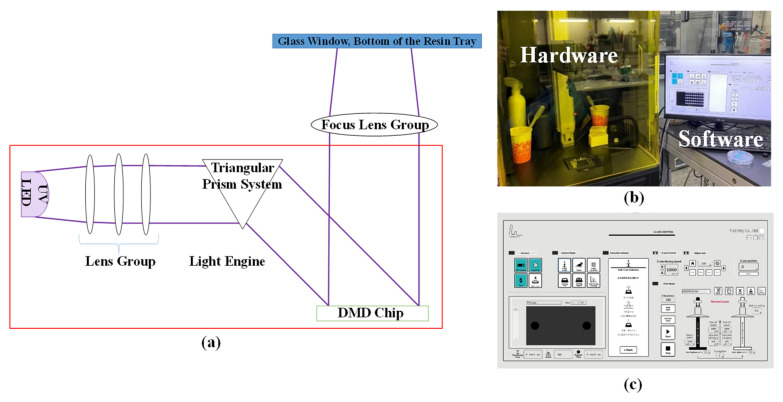
(**a**) Schematic illustration showing the proposed SL additive manufacturing system; (**b**) SL system including hardware and software; (**c**) software control interface.

The equipment used for additive manufacturing is crucial to the success of manufacturing microfluidic devices. In the current study, we selected digital light processing (DLP) stereolithographic (SL) printing (Ulight, FreEntity, Taipei City, Taiwan). The system included a lighting source, a triangular prism system, a digital micromirror device (DMD), a focus lens group, and a resin tray. Figure 1a presents a schematic illustration of this system. Figure 1b illustrates the hardware and software, and Figure 1c shows the software control panel. In this study, we used a standard transparent resin (Durable+, FreEntity, Taipei City, Taiwan), and note that the resin used here belonged to an acrylate system (free radical polymerization) instead of an epoxy system (cationic), because the reactivity of an acrylate system is higher. The lighting source was a UV LED with a wavelength of 405 nm. The digital micromirror device (DMD) included a 1920 × 1080 microarray mirror. The bottom of the resin tray was a glass window. In this configuration, the maximum projected area was 9.6 × 5.4 mm with resolution of 5 μm. Enlarging the projected area would require replacement of the focus lens group and would inevitably lead to lower resolution. Teflon thin film was taped to the glass window on the bottom of the resin tray to prevent attachment of the printed object to the glass window.

### 2.2. Three-Step Printing Process

**Figure 2:**
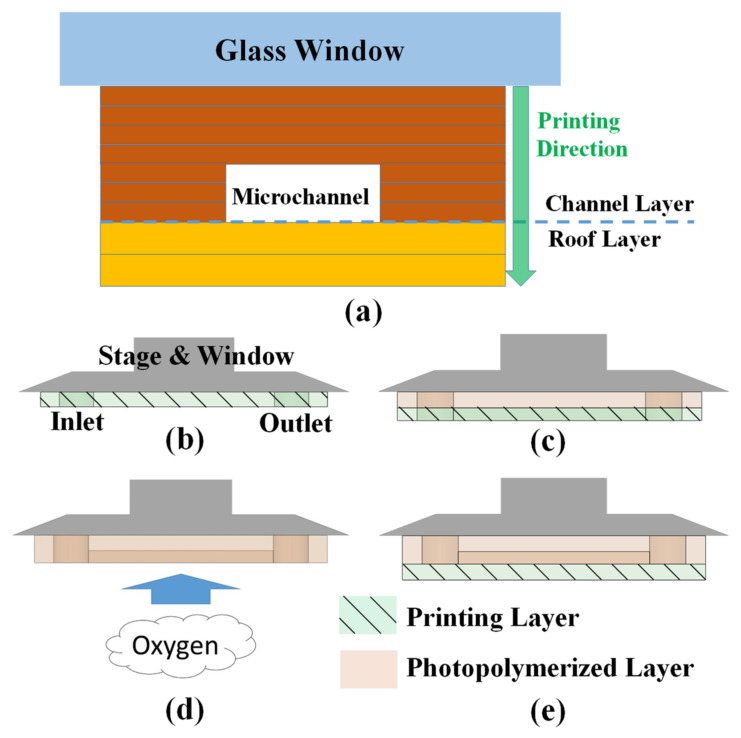
(**a**) Schematic illustration showing a cross-section and the printing process of a monolithic microchannel; (**b**–**e**) the additive manufacturing process, where the green areas with solid lines indicate a printed layer, and the pink areas indicate a photopolymerized layer; (**b**) substrate printing; (**c**) channel layer printing; (**d**) oxygen introduction step to inhibit the photopolymerization of resin inside the microchannel; (**e**) roof layer printing.

Fabricating microchannels with dimensions smaller than 100 μm is challenging because it means printing hollow and micro-scale structures. In the current study, we sought to create a straight microchannel measuring 100 μm in width, 400 μm in height, and 7 mm in length including the inlet and outlet with diameters of 1.15 mm. The overall printing process was implemented in three steps (Figure 2a): (1) manufacturing a microchannel without a roof (“channel layer”); (2) inhibition of resin photopolymerization inside the microchannel by introducing ambient oxygen to overcome the z-overcure error; (3) manufacturing a roof layer (“roof layer”). Figure 2b illustrates the process of printing inlet and outlet reservoirs on the substrate. Figure 2c illustrates the process of printing microchannels. Figure 2d illustrates the inhibition of resin polymerization inside the microchannel by raising the photopolymerized structures up to the ambient air (oxygen) for a certain dwell time. Figure 2e illustrates the process of printing a roof layer to seal the microchannel. Note that the printing thickness of the channel layer is different from the roof layer, because the printing thickness of the channel layer is crucial to the inner surface quality, while the printing thickness of the roof layer is relative to the resin clogging the inside of the microchannels.

**Figure 3:**
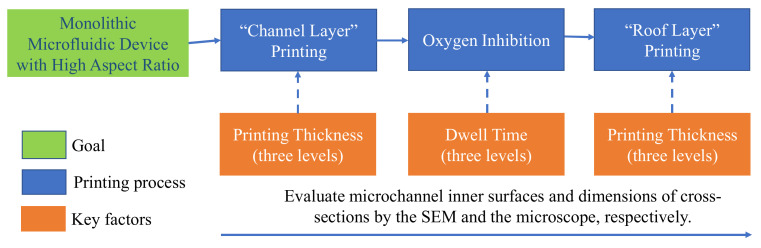
Flow chart showing the overall study process for manufacturing monolithic microfluidic devices including three major factors that each have three levels.

To systematically study the influence of each printing process on the quality of the microchannel, printing thickness and dwell time were studied for the channel and roof layers and oxygen inhibition, respectively, as shown in Figure 3. The goal was to adopt additive manufacturing to create a monolithic microfluidic device with excellent inner surface quality and accurate microchannel dimensions.

### 2.3. Printing Thickness of the Quality of the Microchannels

The dimensional accuracy of the cross-sections and the quality of the inner surface are the two properties with the greatest importance in the fabrication of microchannels. Many microfluidic device applications depend heavily on laminar flow characteristics including inertia microfluidics [21], immunoassay [22], and inertia focusing [23]. Experiments were conducted to assess the influence of printing thickness on the scalloping phenomenon and the quality of the microchannel’s inner surface when fabricating the channel layer and roof layer as shown in Figure 2a. Channel layers were manufactured with a printing thickness of 100, 60, or 20 μm, after which sandpaper (various grades) was used to prepare clear cross-sections for observation and quantification. The roof layer was manufactured with a printing thickness of 110, 120, or 130 μm (note that the printing thickness can be referred to in the equation reported in [18]). A tool microscope (Leica optical microscope, DM series, Allendale, NJ 07401, USA) was used to quantify the dimensional accuracy of the cross-sections, and a scanning electron microscope (SEM, FESEM-6500F, JEOL, Peabody, MA 01960, USA) was used to observe variations in the cross-sections and, particularly, in the surface quality of the microchannel’s inner surface.

### 2.4. Oxygen Inhibition to the Quality of the Microchannels

The experiment results described in Section 3.1. and shown in the Supplementary Materials clearly show that clogging occurs as a result of light penetrating the transparent roof layer to initiate the polymerization of resin within the microchannel, called the z-overcure error, no matter what printing thickness was adopted. We therefore sought to suppress the polymerization capability of resin inside the microchannel before printing of the roof layer. The resin used in this study belonged to acrylated materials, which undergo radical polymerization, easily influenced by molecular oxygen, and result in incomplete curing. Interestingly, Tumbleston et al. reported on continuous liquid interface production to allow the large-scale rapid formation of a polymeric layer at feature resolutions below 100 micrometers. They used an acrylate system resin, and their process relies on the formation of a persistent liquid interface that inhibits photopolymerization through the introduction of oxygen [24]. Following the identical idea of oxygen inhibition to suppress the polymerization of resin located inside the microchannel, we adopted open-air as a strategy. As shown in Figure 2d, newly polymerized structures were suspended in the air, oxygen from ambient air was introduced into the resin inside the unsealed microchannels, and the polymerization capability of resin located inside the microchannel was suppressed. After a set interval, the resin tray was lowered again to print the roof layer as shown in Figure 2e. In the current study, we tested dwell times of 30, 60, and 90 s in a series of experiments.

### 2.5. Microchannels with a High Aspect Ratio

Microchannels with different cross-sections are developed for various applications, and microchannels with a high aspect ratio are preferred for a large number of specific applications. Hung et al. used soft lithography to manufacture a microfluidic device comprising a circular microchamber (40 μm in height) surrounded by multiple narrow channels (2 μm in height) (aspect ratio of 20). Their design was meant to facilitate the localization of cells within the microchamber and create a uniform microenvironment for cell growth [25]. Hood et al. used low-temperature solvent bonding and careful alignment to assemble multi-layer thermoplastic devices for use as a low focusing device (aspect ratio of 100) for liposome synthesis. In that study, the high aspect ratio was meant to enhance throughput, while simultaneously reducing polydispersity [26]. Overall, it is essential to ensure control over the aspect ratio of microchannels, while preserving the ability to employ a high aspect ratio for specific applications. In the current study, we sought to create precise and high-quality microfluidic devices with high aspect ratios from 8:1, 20:1, to 40:1 via a three-step stereolithographic printing process, corresponding to microchannels 800 μm in height and 100 μm in width, 2000 μm in height and 100 μm in width, and 4000 μm in height and 100 μm in width, respectively. Following the devices’ completion, blue dye solution was injected through the microchannels to assess the efficacy of the resulting microfluidic devices.

## 3. Results

### 3.1. Printing Thickness to the Microchannel Quality

**Figure 4:**
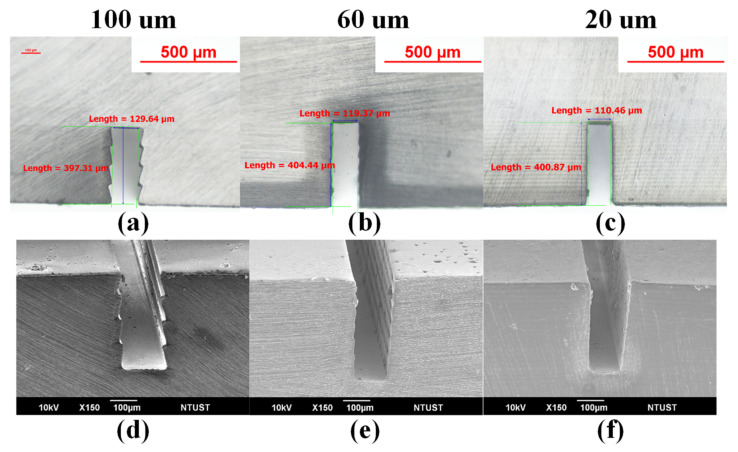
(**a**–**c**) Cross-sections with measurements obtained using a tool microscope; (**d**–**f**) SEM cross-sections corresponding to printing thicknesses of 100, 60, and 20 μm, respectively.

Microfluidic devices require a smooth inner surface and precise microchannel dimensions. Figure 4 presents the printed microchannels with different printing thicknesses. Figure 4a–c present the cross-sections of the printed channel layers with the measurements obtained using a tool microscope. Figure 4d–f present cross-sections of the printed channel layer showing the quality of the channels based on SEM images. The intended width of the microchannel was 100 μm; however, the actual width of the printed device varied as a function of printing thickness as follows: printing thickness of 130 μm (printing thickness of 100 μm), 120 μm (printing thickness of 60 μm), and 110 μm (printing thickness of 20 μm). From Figure 4d–f, it is clear that decreasing the printing thickness resulted in a smoother inner surface, whereas increasing the printing thickness resulted in a stair-like inner surface (i.e., scalloping phenomenon), which could introduce irregular flow patterns. The printing time varied as a function of printing thickness as follows: 100 μm (9’40”), 60 μm (10’18”), and 20 μm (13’43”). As shown in Figure 4, decreasing the printing thickness from 100 to 20 μm in the channel layer increased the processing time by 40%; however, it greatly improved the quality of the inner surface and the dimensional accuracy of the width. 

Here, we describe the preliminary experimental results, where only the two-step printing process was adopted by executing the printing channel layer and printing roof layer. After completion of the channel layer shown in the Figure 4, the roof layer was printed continuously to seal the microchannel. The roof layer was applied in three printing thicknesses (i.e., 110, 120, and 130 μm), and the results are shown in Supplementary Materials Figure S1. It is clear that the dimensions of the microchannel were not correct due to the partial clogging. Then, we sought to suppress the clogging (or called the z-overcure error due to the resin polymerization within the microchannel when printing the roof layer) by reducing the exposure energy (either by reducing the lighting intensity or reducing the exposure time). Unfortunately, this approach actually made the roof layer collapse due to the fact that there was insufficient energy to polymerize the roof layer (see Supplementary Materials Figure S2) and proved that printing a micro and hollow structure is challenging. We also observed slight microchannel swelling (see Supplementary Materials Figure S1d–f), attributable to the process by which excess resin was removed after printing the roof layer. Basically, this involved injecting ethanol solution into the channels and then soaking the entire printed device into an ethanol solution under ultrasonication for 30 s. At the time of cleaning (i.e., prior to final baking at 60–80 °C for 1 h), the microfluidic device had not undergone complete polymerization. As a result, the injection of the ethanol solution caused the microchannel side walls to deform slightly. It is clear that neither reducing the printing thickness nor reducing the printing energy can create a microchannel with accurate dimensions and a smooth inner surface, so we added an oxygen inhibition step into our manufacturing process.

### 3.2. Oxygen Inhibition on the Microchannel Quality

**Figure 5:**
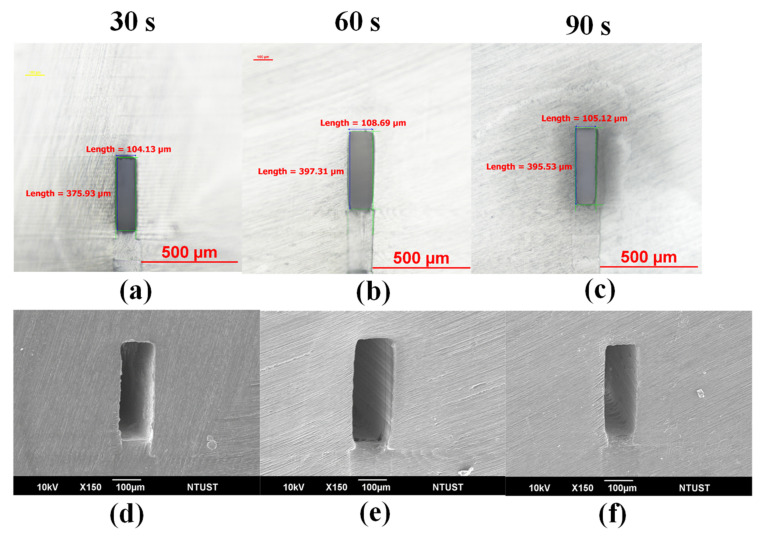
(**a**–**c**) Cross-sections with measurements obtained using a tool microscope; (**d**–**f**) SEM cross-sections corresponding to dwell times of 30, 60, and 90 s, respectively.

Reducing the printing thickness was shown to improve the microchannel quality; however, the aforementioned clogging remained a serious issue, regardless of the thickness of the roof layer. As described in Section 2.4., we sought to overcome this issue by interrupting the printing process by introducing oxygen. Figure 5a–c present cross-sections of the printed channel layer with measurements obtained using a tool microscope. Figure 5d–f present SEM cross-sections of the printed channel layer corresponding to dwell times of 30, 60, and 90 s. These results clearly demonstrate the effectiveness of the oxygen introduction to suppress the resin polymerization inside the microchannel during the roof printing. Here, the depths of the channels were very close to the intended depth of 400 μm, which varied only slightly as a function of dwell time: 376 (oxygen inhibition time of 30 s, 397 (oxygen inhibition time of 60 s), and 395 μm (oxygen inhibition time of 90 s). Overall, the width of the microchannels ranged from 104 to 108 μm. Clearly, the ingress of oxygen between printing stages (from layer printing to roof printing) greatly improved the quality of the resulting microchannels in terms of dimensional accuracy and being clogging free. A dwell time of 60 s was sufficient to create channels very close to the intended depth; therefore, we adopted a dwell time of 60 s for subsequent experiments.

## 4. Monolithic Microchannels with High Aspect Ratios

**Figure 6:**
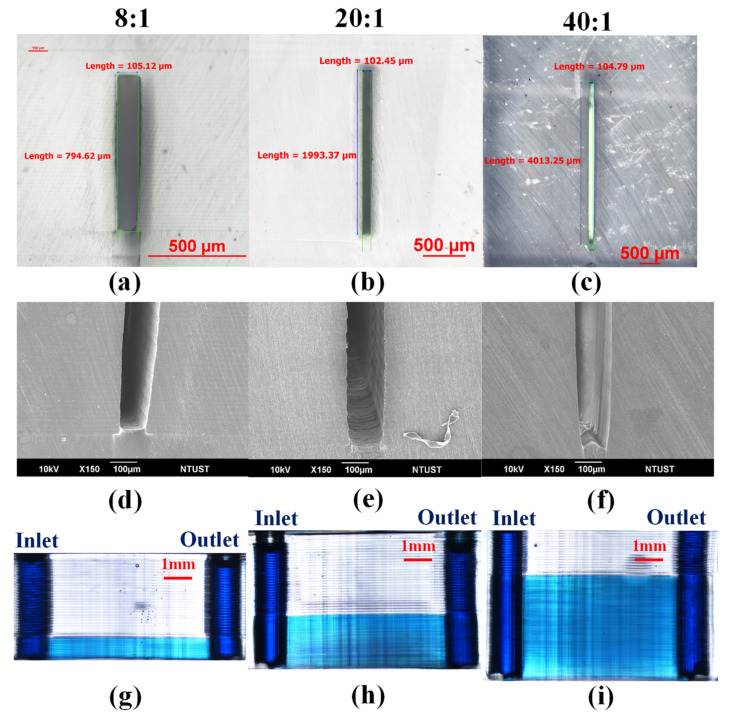
(**a**–**c**) Microchannels with cross-sectional areas of 795μm x 105μm (8:1), 1993μm × 102μm (20:1), and 4013μm × 105μm (40:1); (**d**–**f**) Partial SEM cross sections corresponding to aspect ratios of 8:1, 20:1, and 40:1; (**g**–**i**) side view of the microfluidic devices after injection of blue dye solution.

We fabricated microchannels with high aspect ratios of 8:1, 20:1, and 40:1 using the parameters derived in previous sections as follows: channel layer printing thickness (20 μm), roof layer printing thickness (110 μm), and dwell time (60 s). Figure 6a–c present cross-sections with measurements obtained using a tool microscope. Note that the dimensions of the printed microchannels were 795 × 105 μm (aspect ratio = 8:1; manufacturing time = 20’04”), 1993 × 102 μm (aspect ratio = 20:1; manufacturing time = 33’46”), and 4013 × 105 μm (aspect ratio of 40:1; manufacturing time = 57’06”). Figure 6d–f present SEM cross-sections corresponding to aspect ratios of 8:1, 20:1, and 40:1. Note that slight swelling was observed in all three cases. Figure 6g–i, respectively, show the side view images of the microfluidic devices after the injection of blue dye solution. Note that even the microchannel with an extremely high aspect ratio (40:1) was fully operable. The microfluidic devices printed in this study were too small (device length: 9.6 mm, device width: 5.4 mm, and device height: 5.5 mm) for post-processing (polishing); therefore, printing marks remained observable in all three cases.

While other research groups studied the material composition of resin for successfully manufacturing microchannels by stereolithography printing, the experimental results shown above clearly demonstrated another path for manufacturing microchannels by adopting a three-step printing process with stereolithography printing. This method can not only be used to rapidly manufacture a polymeric microchannel with dimension close to 100 μm but can also be used to manufacture microchannels with extremely high aspect ratios up to 40:1. The most critical step is the oxygen inhibition, because it resolves a challenging problem of resin clogging inside the polymerized microchannel while maintaining the dimensional accuracy of the microchannel. 

## 5. Conclusions

Additive manufacturing has attracted considerable attention for its extensive customizability and its efficiency in terms of time and manufacturing waste. Numerous researchers have used additive manufacturing to create microfluidic devices; however, manufacturing microchannels smaller than 500 μm still remains challenging. In the current study, we sought to use SL additive manufacturing with a three-step printing strategy for the fabrication of 100 μm microchannels with a smooth inner surface and high dimensional accuracy. Extensive experiments led to the following conclusions: (1) Stereolithographic printing (SL) can be used to successfully create monolithic polymeric microfluidic devices with dimensions close to 100 μm. The three-step printing process included manufacturing a microchannel without a roof (“channel layer”), inhibition of resin photopolymerization inside the microchannel by oxygen introduction, and sealing of the microchannel by manufacturing a roof layer (“roof layer”). (2) Printing thickness must be reduced to significantly minimize the scalloping phenomenon as well as to seek the creation of microchannels with high inner surface quality and accurate dimensions. (3) Oxygen inhibition is an efficient approach to significantly improving dimensional accuracy by suppressing resin polymerization within the microchannel when printing the roof layer. The method reported herein is quite simple, requiring only the suspension of the polymerized channel layer in the air for a while. Note that this oxygen inhibition phenomenon only applied to the resin of an acrylate system, which is the most common type of resin for an SL printing process. (4) By following the parameters reported previously, a microchannel with an extremely high aspect ratio of 40 was successfully created within one hour and demonstrated with the injection of blue dye solution.

## Data Availability

Will provide upon request.

## Supplementary Materials

The following are available online at https://www.mdpi.com/article/10.3390/polym13213750/s1, Figure S1: Cross sections with measurements obtained using a tool microscope and SEM; Figure S2: Collapsed roof layer due to the reduced exposure energy.
