# Supplementary material for: Using Stereolithographic Printing to Manufacture Monolithic Microfluidic Devices with an Extremely High Aspect Ratio
Source: Polymers (Basel). 2021 Oct 29;13(21):3750. doi: 10.3390/polym13213750 (PMC8587111; doi:10.3390/polym13213750)
Supplement: Supplementary file 1 [file polymers-13-03750-s001.zip › polymers-1412856-supplementary.pdf]

Supplementary Materials

Using Stereolithographic Printing to Manufacture Monolithic Microfluidic Device with Extremely High Aspect Ratio

Pin-Chuan Chen [1,2], Po-Tsang Chen [1], Tuan Ngoc Anh Vo [1]

[1]Department of Mechanical Engineering, National Taiwan University of Science and Technology, Taiwan.
[2]High Speed 3D Printing Research Center, National Taiwan University of Science and Technology, Taiwan.

**Corresponding author: Prof. Pin-Chuan Chen*

E-mail: pcchen@mail.ntust.edu.tw *Phone: +88627376456. Fax: +88627376460*

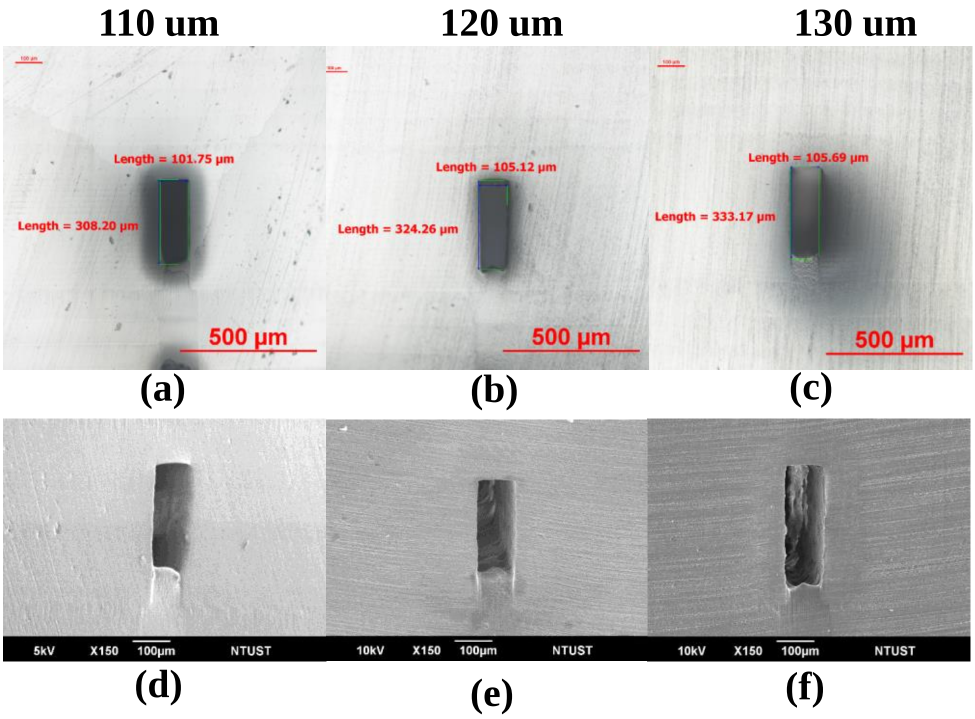

Figure S1: (a) to (c) cross sections with measurements obtained using a tool microscope; (d) to (f) SEM cross sections corresponding to printing thicknesses of 110µm, 120µm, and 130µm, respectively. After completion of the channel layer, the roof layer was printed continuously to seal the microchannel. The roof layer was applied in three printing thicknesses (110µm, 120µm, and 130µm). The intended depth of the microchannel was 400 µm; however, the actual depth varied as a function of printing thickness, as follows: 308µm (printing thickness of 110µm), 324µm (printing thickness of 120µm), and 333µm (printing thickness of 130µm).

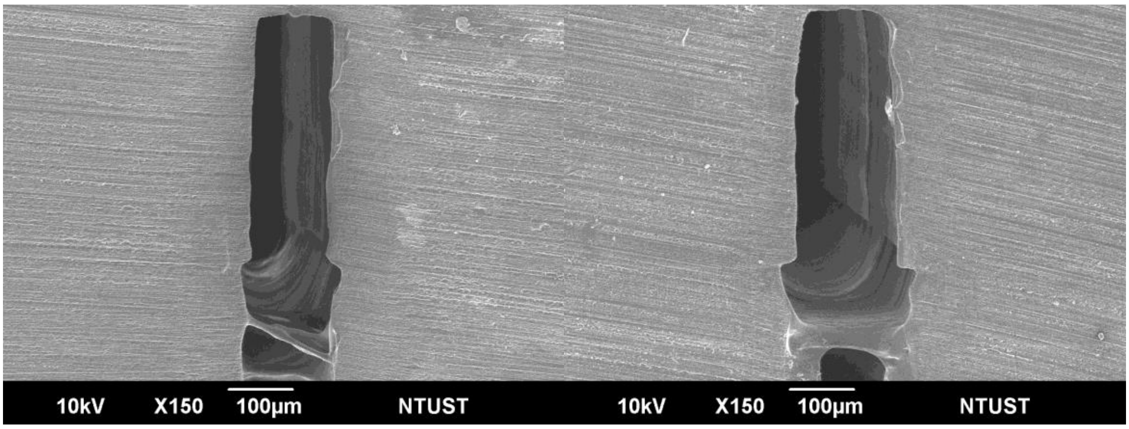

(a) **(b)**

Figure S2: (a)(b) both figures showed the collapsed roof layer due to the reduced exposure energy (either reducing the lighting intensity or reducing the exposure time).
